# A Case of Excision of the Hip-Joint for Morbus Coxarius

**Published:** 1857-06

**Authors:** R. A. Kinloch

**Affiliations:** Surgeon to the Roper Hospital, and Lecturer on Surgery in the Charleston Summer Medical Institute


### *A Case of Excision of the Hip Joint for Morbus Coxarius, with remarks upon the propriety of such an operation, and a summary account of the recorded cases up to the present time.* By R. A. Kinloch, M. D., Surgeon to the Roper Hospital, and Lecturer on Surgery in the Charleston Summer Medical Institute.

John McAllen, a native of Ireland, aged twenty, a laborer by occupation, was admitted into the Roper Hospital on the 2d of April, 1856. Two years previous he began to suffer from vague, undefined pain about the lumbar region and the hip joint of the right side. From time to time he had been compelled to lie up for a short period, but never, until nine months ago, had he been sufficiently unwell to be obliged to give up his usual avocations. More lately his attention had been attracted by a decided swelling beginning to manifest itself upon the front of his thigh. This had gone on increasing for several weeks, until it had attained its present volume. There was also a disposition, lately, for the thigh to become flexed upon the pelvis, and now the limb could not be entirely straightened. Upon examination of the patient, who was emaciated and presented a marked scrofulous appearance, I found a large fluctuating swelling, evidently sub-fascial, occupying nearly the whole anterior part of the middle third of the right thigh. The thigh was slightly flexed upon the pelvis, and the knee and foot turned a little in; the limb, generally, was emaciated. There was likewise a small swelling over the nates, behind and lower than the trochanter: this, like the larger swelling, did not in any way disappear on pressure. The swelling upon the thigh was what had given the patient most anxiety, and induced him to come to the hospital. He only wanted something done for this, as he felt well enough, as yet, to go about, and for aught else would not have been disposed to lie up. The diagnosis was at once made out as Morbus Coxarius. The swellings were considered to be chronic abscesses, but at first I could not determine if they communicated with the joint. The disease of the joint did not seem to have progressed very far, as the patient walked very regular, and the joint appeared very well: no grating could be discovered on manipulation, nor did extensive movement give much pain. I delayed opening the abscesses for several days, in order to watch a little the constitutional power of my patient, and further, too, to have my diagnosis more certain. I prescribed for him cod liver oil ℥ss. with wine ℥ij., three times a day, and an anodyne at night. April 10th, the abscess of the thigh having materially increased in size, and the patient complaining of the feeling of tension, I made a kind of valvular opening at the dependant part, on the outer side of the sartorius muscle, and evacuated a large quantity of thin pus. April 22d I punctured the abscess over the nates, and, subsequently to this, both abscesses having refilled, were again opened on several occasions. May 4th, distinct fluctuation was discoverable higher up, behind the trochanter. A puncture was made here, and considerable pus flowed away, mixed, it appeared, with synovial fluid. Consequent upon this, there supervened considerable irritative fever, with gastric disturbance and great restlessness. The oil was now discontinued, and p. gum opii, gr. j., with a glass of wine, given every three hours. The distressing symptoms were modified after a couple of days, and the opium was then only given at bed time. The discharge from the last abscess was now very copious and offensive. I had expressed a gloomy prognosis to some friends of the patient, and on this account I was applied to, on May 10th, to give him a discharge from the hospital, his friends preferring that he should die at home. At their earnest solicitation, a few days after, I consented to visit him privately. At the expiration of a few days, I was much pleased to notice that a decided improvement had taken place under a free allowance of good wine, with nourishing diet, and opiates given when required. The discharge, though, continued profuse, and there were many evidences of extensive disease of the joint. Once or twice, when at the hospital, he heard me say something about dead bone and the persistence of the discharge. He now wanted to know if I could not in any way take out the dead bone, saying that he would submit to any operation that promised success. Eight or ten days elapsed, and finding that the discharge was as profuse as ever, moreover that ugly bed-sores were about appearing, I began to think that excision of the joint might offer a little chance He seemed strong enough to stand an operation quickly performed, and this appeared the only alternative left. I then fairly stated to him my opinion, and told him how slight a chance the operation afforded; that it might hasten his end. He decided that the attempt should be made. I assumed the responsibility; but I determined that first I would explore the condition of the joint by making a free opening upon it through the cavity of a large abscess, just above the trochanter, and over the head of the femur. To open this abscess freely, I conceived to be proper practice, even though I did not intend to resect the joint. It would be but the carrying out the improved plan of Mr Gay for treating suppurating joints. If the capsule of the joint was found open, and the head of the bone caried, then I would proceed to resect. Previous to commencing my incisions, I had decided, by the test of Nélâton, that the head of the femur *was still in the acetabulum.* On the 31st May, 1856, my patient having been brought under the influence of ether, (I used ether then instead of chloroform for the first and last time, at the suggestion of a friend; the patient took fully a pound before he became affected,) was turned slightly upon the left side, and held in that position by assistants. I then thrust the point of a small amputating knife a little in front of the base of the trochanter, and carrying it upwards and outwards, and then downwards and backwards, formed a semilunar incision encircling the trochanter, and consequently had a sort of semilunar flap with the convexity upwards. The knife went easily through the walls of the abscess alluded to above, and exposed its entire cavity. The finger passed in, and to the bottom of the wound, discovered plainly considerable destruction of the capsule of the joint, and also the head of the bone, still in the acetabulum, but quite rough, and partially destroyed by caries; the brim of the acetabulum, too, could be felt considerably diseased. Under these circumstances, I conceived it best to proceed and resect the head of the femur. The point of the knife was accordingly passed across the portions of the capsule yet entire. My assistant then flexed and adducted the thigh, and the round ligament not holding, the head was thrown out of the cavity, and then forced as much as possible through the external wound. I next passed the chain-saw behind the head and neck, and quickly divided the bone above the trochanters. Proceeding to examine the acetabulum, I was shocked at the extent of the disease. The brim was rough and crumbling, and there was an extensive perforation of the floor. With the gouge forceps I took away some portions of the brim, but soon desisted, as I felt it impossible to take away anything like the whole of the diseased structures. The wound was brought together by a few sutures, the lower end only kept open by a little lint to facilitate the exit of the discharge, the patient removed to bed, and the limb extended and kept steady by pillows. Not reacting as well as I desired, I ordered brandy freely until my afternoon visit, also a full dose of opium. In the afternoon his condition seemed better; he had warmth of skin and a fair pulse. The brandy was continued according to the indication during the night, but upon my morning visit I was sorry to discover a greater disposition to collapse. From this time he refused to respond to the most active internal and external stimulation, but sank and died, not quite thirty hours after the operation. I was not able to examine the body after death.

*Remarks.*—One of the prominent features of modern surgery is its conservatism. This is especially evidenced by the number of operations now performed for the excision of joints, in consequence either of injuries or diseases which formerly seemed to necessitate amputation. We may say now, with reference to the joints of the upper extremity, that good surgery always calls for excision, where amputation is the only alternative. Even though excision should offer a little more risk to life—which, however, cannot be affirmed—it is rendered attractive and preferable when we think of the advantages of possessing a useful member. With the larger joints of the lower extremity, the rule of practice is not so strikingly clear nor so certainly fixed; or, at any rate, there exists more controversy as to the expediency of excision in injuries and diseases of these structures. To decide correctly, it is evident that truth must be elicited on two important points: on the comparative danger to life from amputation, and from excision, and the utility of the limb after successful excision, when compared to an artificial substitute. To determine these points, the excisions of the several joints must be considered separately, and compared with the several amputations which in practice may become their substitutes. Thus, with regard to the hip joint, the excision must be contrasted with the amputation through the joint; while with the knee and ankle joints, the excisions are to be contrasted with amputations of the thigh and leg. It will be perceived, then, that the question is not precisely the same with regard to the three large joints of the lower extremity. When considering amputation or excision of the hip joint, we must decide, first, which of the two operations offers less risk to life; and, secondly, the probable usefulness of the limb after excision. We cannot well speak of the utility of the member as compared with a substitute, because no contrivance that we know of is worthy of such a name. We have only to consider whether the limb, after even successful excision, is not worse than useless—is not a positive incumbrance. If it be an incumbrance, the merits of excision of the joint would have to rest upon its claim of occasioning less danger to life. If such claims could not be substantiated, then excision would be bad surgery; amputation would be correct practice. But as to the exsection of the knee and ankle joints, the danger of these, as compared with the respective amputations of the thigh and leg, must first be weighed, and then a decision must be further founded upon the known usefulness of the limb, in each case, after successful exsection, as contrasted with its usefulness after successful amputation, when there has been provided an artificial leg or foot.

These are the two prominent questions, when considering the propriety of exsections of the hip, knee, or other joints, as substitutes for amputation. But it must not be forgotten that there is another question of paramount importance which comes up in many cases of diseased joints, viz.: the propriety of resorting to any operative procedure. This is the first question to be determined, when we have to deal with diseases of the joints considered constitutional in their character; or when, along with the diseased joint, we have functional or organic disease of some important organ. The rule of surgery is very plain as to the latter contingency. Very rarely would an amputation or excision, of the kind we are considering, be a justifiable procedure in a patient with diseased lungs or kidneys. But the rule is perhaps not so fixed in reference to the first contingency. In regarding the topical manifestation, we may be directed back to some general disease, some abnormal *blood crasis*. There may be some practitioners who, carried away with a transcendental humoralism, look only to this, and can see no philosophy in active local interference. Others—and we must think these the more rational—cannot see why the common rules of surgery should be departed from, when dealing with the effects of the disease in a particular texture, because the local changes may be obscurely traceable to faults in the general nutrition; admitting the *blood crasis*, they cannot, on this account, forget that the local affection *may be the source of increased potency to the general disease, and likewise that it may be independently progressive.*

How are we to discover truth in reference to these debatable points?

To reason *à priori*, we may approximate it, by applying the common principles of surgical science, of analogy, and of common sense; but to reach it definitely, we must be guided by experience alone—we must lean upon the solid basis of statistical data.

First, then, to glance at these principles, and then at the statistical data, so far as this may bear upon the propriety of excision of the hip joint in "Morbus Coxarius." The pathology of this affection I will say, in a few words, is scrofulous caries, beginning, in the greater portion of cases, in the head of the femur, and attended, sooner or later, where disease progresses, with destructive ulceration of the cartilages and the ligamentous tissue of the joint, going on finally to caries of the floor and brim of the acetabulum, and the formation of abscesses about the joint, which may at last communicate externally, and in some instances even open within the pelvis. The progress of the disease may be in one of several directions. Sometimes it is favorable, a limit taking place to the destructive process, successful attempt at new ossific production, with gradual cessation of the discharge results, the joint becoming anchylosed; or a further destructive progress may have first eventuated in the displacement of the head of the bone from its proper cavity, and its subsequent fixation upon the ilium, the acetabulum recovering from disease; or, lastly, an actual separation of the caried head of the femur from the shaft, and its subsequent exfoliation, may prove the beginning of a hopeful result. At other times, the progress is in the more fearful direction: the patient becomes worn out, and dies from hectic, the local disease having extended its ravages, and the abnormal *blood crasis* having become more confirmed; often, too, other and more important organs have suffered from destructive disease.

Now, in regard to the treatment of such an affection, the principles of surgery should be clear and determined. The general *blood crasis* must be corrected, if possible, and its morbid local manifestation controlled. The means for fulfilling the first indication will assist in fulfilling the second; but these must not be relied on alone; general and local treatment must go together. The kind of local treatment, however, must be determined, in a great measure, by the degree of progress the disease has made. In the early stages, the local treatment might have for its paramount object the controlling of increased local vascular excitement; but at a later period the indication is not so simple—for we have to deal with superadded sources of disease, in the shape of purulent accumulation, and dead cartilage and bone. The principles of surgery, now, would seem to call for more active local interference—for we know of but one way of dealing with dead bone: *it must be removed—for it is, to all intents and purposes, a foreign body.* We hesitate not to carry out this principle in caries or necrosis attacking most other parts of the skeleton, whether connected or not with strumous disease. The indication is ever the same, and we should refrain from carrying it out only where the operative procedure necessary for so doing would be almost certainly destructive of life, as in the caries of the spinal column, for example.

Does the operative procedure for the removal of the necrosed bone, in "Hip Disease," involve an amount of risk sufficient to deter us from resorting to it? I will answer this hereafter.

The same kind of *blood crasis* often presents us with local diseased manifestations in other structures and organs : in the lymphatic glands, the breast, the testicle. If disease progresses far enough in these organs to threaten life by hectic, in consequence of the wasting discharge and the irritative action engendered and kept up by the presence of diseased, weak, and dying tissues, does any principle of surgery advise against operative interference because of scrofulosis? I think not. May I not, then, make bold to say, that the principles of science, analogy, and common sense, offer no objection to surgical interference in the disease under consideration, but rather urge its propriety?

If we appeal in the same direction for a decision as to the choice of the two operations above referred to, there would seem to be even less room for disputation here than there is with the previous question. The important parts necessarily cut through in amputation, the degree of shock resulting, the extent of the wound made by the procedure, enable us at once to pronounce upon the likelihood of a much greater attendant fatality than would follow the comparatively small operation of excision.

For information as to the utility of the limb after successful excision, we can assuredly rely upon experience alone. The result of this experience I will show in presenting the statistical data, and then, too, we shall see what reliance can be placed upon the conclusions already arrived at by *á priori* reasoning.

Mr. Charles White, of England, in 1769, seems to have first suggested the excision ef the Hip Joint; he, however, never practiced the operation. Schmalz, a Saxon surgeon, in 1816, first removed the head of the femur for "Morbus Coxarius". He did not, though, practice resection of the bone, for the head was found already separated from the neck; nevertheless, his operation is justly made to rank under this head. In England, the operation was first performed by Mr. Anthony White, in 1818; and it attracted much attention because of its novelty and its successful result. Mr. Hewson, of Dublin, repeated the operation in 1823, and Sir Benjamin Brodie in 1836. Besides Sir B. Brodie's case, we find, after Howson's, an account of some seven operations by Heim, Schlitching, Kluge, Vogel, and Textor. The operation, however, passed into a kind of oblivion, until Mr. Fergusson, in 1845, lent it the authority of his distinguished name. He deserves the credit of reviving it in Great Britain, for it has since been practiced by many of the best surgeons in the metropolis of England, and has there become a recognized procedure. Elsewhere it has been little resorted to.—*Charleston Medical Journal & Review.*

